# Stroke risk and outcomes in patients with chronic kidney disease or end-stage renal disease: Two nationwide studies

**DOI:** 10.1371/journal.pone.0191155

**Published:** 2018-01-12

**Authors:** Yih-Giun Cherng, Chao-Shun Lin, Chun-Chuan Shih, Yung-Ho Hsu, Chun-Chieh Yeh, Chaur-Jong Hu, Ta-Liang Chen, Chien-Chang Liao

**Affiliations:** 1 Department of Anesthesiology, Shuang Ho Hospital, Taipei Medical University, New Taipei City, Taiwan; 2 Department of Anesthesiology, School of Medicine, College of Medicine, Taipei Medical University, Taipei, Taiwan; 3 Department of Anesthesiology, Taipei Medical University Hospital, Taipei, Taiwan; 4 Anesthesiology and Health Policy Research Center, Taipei Medical University Hospital, Taipei, Taiwan; 5 School of Chinese Medicine for Post-Baccalaureate, College of Medicine, I-Shou University, Kaohsiung, Taiwan; 6 Program for the Clinical Drug Discovery from Botanical Herbs, Taipei Medical University, Taipei, Taiwan; 7 Department of Nephrology, Shuang Ho Hospital, Taipei Medical University, New Taipei City, Taiwan; 8 Department of Nephrology, College of Medicine, Taipei Medical University, Taipei, Taiwan; 9 Department of Surgery, China Medical University Hospital, Taichung, Taiwan; 10 Department of Surgery, University of Illinois, Chicago, United States of America; 11 Department of Neurology, Shuang Ho Hospital, Taipei Medical University, New Taipei City, Taiwan; 12 School of Chinese Medicine, College of Chinese Medicine, China Medical University, Taichung, Taiwan; Kaohsiung Medical University Hospital, TAIWAN

## Abstract

**Background and aims:**

Because the risk and outcomes of stroke in patients with chronic kidney disease (CKD) or end-stage renal disease (ESRD) were unclear, we evaluated these risks using a retrospective cohort study and a nested cohort study.

**Methods:**

We used Taiwan’s National Health Insurance Research Database to identify 1378 patients aged ≥20 years who had ESRD in 2000–2004. An age- and sex-matched CKD cohort (n = 5512) and a control cohort (n = 11,024) were selected for comparison. Events of incident stroke were considered as outcome during the follow-up period in 2000–2013, and we calculated adjusted hazard ratios (HR) and 95% CIs of stroke associated with CKD or ESRD. We further used matching procedure with propensity score to estimate the risk of stroke for control group, CKD patients, and EDRD patients. A nested cohort study of 318,638 hospitalized stroke patients between 2000 and 2010 also was conducted to analyze the impact of CKD and ESRD on post-stroke mortality.

**Results:**

Before propensity-score matching, the incidences of stroke for controls, CKD patients and ESRD patients were 6.57, 13.3, and 21.7 per 1000 person-years, respectively. Compared with control group, the adjusted HRs of stroke were 1.49 (95% CI, 1.32–1.68) and 2.39 (95% CI, 1.39–2.87) for people with CKD or ESRD respectively, and were significantly higher in both sexes and every age group. After propensity-score matching, the HRs of stroke for patients with CKD and ESRD were 1.51 (95% CI 1.24–1.85) and 2.08 (95% CI 1.32–3.26), respectively, during the follow-up period. Among hospitalized stroke patients, adjusted rate ratio (RR) of post-stroke mortality in CKD and ESRD cohorts were 1.44 (95% CI, 1.33–1.56) and 2.62 (95% CI, 2.43–2.82) respectively compared with control.

**Conclusions:**

CKD and ESRD patient groups thus faced significantly higher risk of stroke and post-stroke mortality. Risk factor identification and preventive strategies are needed to minimize stroke risk and post-stroke mortality in these vulnerable patient groups.

## Introduction

Chronic kidney disease (CKD) and its associated neurological complications have been recognized as a major global health concern for decades [1]. The occurrence of cerebrovascular disease in patients with CKD is frequently due to both conventional and unconventional cardiovascular factors [2–4]. Increased chronic inflammation and oxidative stress, decreased nitric oxide production, and hyperhomocysteinaemia that might induce endothelium dysfunction, platelet aggregation, and vascular injury contribute to neurological insults in CKD patients [2,3].

Uremia-associated risk factors such as uremic toxins, sodium and fluid accumulation, decreased erythropoiesis, albuminuria, impaired calcium and phosphate metabolism, and hyperparathyroidism also increase cerebrovascular disorders among CKD patients [2,3]. Previous studies found increased risk of stroke when non-dialysis-dependent CKD progressed to end-stage renal disease (ESRD) [5]. However, differences in risk factors, stroke type, severity, and post-stroke outcomes between CKD and ESRD were not well investigated. Recent improvements in dialysis techniques prolong lifespans, but these uremic patients develop atherogenic changes, endothelial dysfunction, and vascular calcification [2,3,6]. Due to these changes, stroke becomes a major health issue for patients with impaired renal function [1,2,6,7].

Although the incidence, prevalence, risk factors, and adverse outcomes of stroke in patients with CKD or ESRD have been reported, these studies are limited by small sample sizes [8–16], lack of control group [9] and matching procedure [8,10–12], inadequate adjustment for potential confounders [15,16], or focus on either CKD or ESRD population [8–16]. Yet the comprehensive features and stroke risk differences relating to CKD and ESRD were unclear, and renal illness’s impacts on post-stroke outcomes were not known. Using Taiwan’s National Health Insurance Research Database, we conducted two nationwide studies to evaluate stroke risk and mortality in patients with CKD or ESRD.

## Methods

### Source of data

The National Health Insurance Program was implemented in March 1995 and now covers more than 99% of 23 million Taiwan residents with comprehensive health services. This database records patients’ date of birth, sex, residence, and levels of insurance fees as well as medical records including physician diagnoses, prescriptions, treatment, medical expenditures, and examination records for all medical services, including outpatient care, emergency treatment, hospitalization, traditional Chinese medicine, and dental care [17,18].

### Ethical approvals

Insurance reimbursement claims used in this study were from Taiwan’s National Health Insurance Research Database, which was available for public access. To protect personal privacy, the electronic database was decoded with patient identifications scrambled for further public access for research. According to Taiwan National Health Research Institutes regulations, informed consent is not required because of the use of decoded and scrambled patient identification. However, this study also was evaluated and approved by the joint institutional review boards of Taipei Medical University in Taipei (TMU-JIRB-201509050) and E-Da Hospital in Kaohsiung, Taiwan (EDA-JIRB-2014012). The data underlying this study is from the National Health Insurance Research Database, which has been transferred to the Health and Welfare Data Science Center. Interested researchers can obtain the data through formal application to the Health and Welfare Data Science Center, Department of Statistics, Ministry of Health and Welfare, Taiwan (http://dep.mohw.gov.tw/DOS/np-2497-113.html).

### Study design

To investigate the risk of stroke in patients with ESRD, we conducted a retrospective cohort study and identified 1378 patients aged ≥20 years with ESRD (defined as receiving regular hemodialysis therapy 2–3 times per week) from the database of a sample of one million beneficiaries in 2000–2004, excluding those receiving peritoneal dialysis. Using frequency matching procedure, we selected 5512 CKD patients (with at least three occasions when physicians’ primary diagnosis was CKD; case-control ratio = 1:4) and 11,024 controls (without any history of CKD or ESRD; case-control ratio = 1:8) from the same database as comparison cohorts during the same index period. For reducing the confounding bias, we further used exact matching procedure with propensity score (case-control ratio = 1:1) to select more appropriate ESRD patients (n = 320), CKD patients (n = 2758), and control group. These three cohorts had no history of stroke and were followed from 2000 until the end of 2013 to identify any new event of stroke. In the Study I, we used this representative sample of one million beneficiaries that allow us to analyze the long-term risk of stroke for control group, CKD patients and ESRD patients during the follow-up period. The purpose of Study I (the retrospective cohort study) was to investigate the risk of stroke for patients with and without CKD or ESRD.

Using reimbursement claims from the same National Health Insurance Research Database, we also conducted a nested stroke cohort study (Study II) of all stroke patients with hospitalization care between 2000 and 2010 across Taiwan. The Study II included large sample of 318,638 newly diagnosed hospitalized stroke patients aged ≥20 years that allow us to analyze enough death cases in the short-term period after stroke. The purpose of Study II is to investigate the impact of history of CKD and ESRD on the 30-day post-stroke mortality. Thirty-day in-hospital mortality after stroke was compared in stroke controls (stroke patients with no histories of CKD or ESRD), stroke patients with CKD history, and stroke patients with ESRD history.

### Criteria and definitions

Patient’s low-income status was verified by the Ministry of Health and Welfare. The levels of residence urbanization were categorized according to population density, which was calculated by dividing population (persons) by area (in square kilometers) for each administrative unit of Taiwan. The population density was divided into quartiles and categorized into the lowest quartile, moderate (the second and third quartile), and high urbanization (the fourth quartile). We used *the International Classification of Diseases*, *Ninth Revision*, *Clinical Modification* (ICD-9-CM) to define physician diagnoses of diseases including stroke (ICD-9-CM 430–438), hypertension (ICD-9-CM 401–405), anemia (ICD-9-CM 280–285), ischemic heart disease (ICD-9-CM 410–414), diabetes (ICD-9-CM 250), mental disorders (ICD-9-CM 290–319), chronic obstructive pulmonary disease (ICD-9-CM 490–496), heart failure (ICD-9-CM 428), hyperlipidemia (ICD-9-CM 272.0, 272.2, 272.4), traumatic brain injury (ICD-9-CM 800–804, 850–854), liver cirrhosis (ICD-9-CM 571), atrial fibrillation (ICD-9-CM 427.31), peripheral vascular disease (ICD-9-CM 440, 443), and epilepsy (ICD-9-CM 345). The administration code was used to define renal dialysis (D8, D9). This study also considered use of anticoagulants, anti-hypertensive agents, and statin cardiovascular drugs. Anti-hypertensive medications included diuretics (such as Hydrochlorothiazide, Furosemide, Spironolactone), ACEI (such as Benazepril, Enalapril, Cilazapril, Fosinopril, Lisinopril, Moexipril, Quinapril, Ramipril), ARB (such as Candesartan, Eprosartan, Irbsartan, Losartan, Olmesartan, Valsartan), beta-blockers (Atennolol, Carvedilol, Labetalol, Metoprolol), CCB (Amlodipine, Cardil, Felodipine, Lacidipine, Nicardipine Nifedipine, Nisoldipine), and alpha-Blockers (Doxazosin, Prazosin, Terazosin). Anticoagulants included Warfarin, Dabigatran, Rivaroxaban, Apixaban, Heparin, Dalteparin, Enoxaparin, Nadroparine, Tinzaparin, Clopidogrel, Ticlopidine, Aspirin, Dipyridamole, Epoprostenol, Iloprost, Eptifibatide, Eptifibatide, Tirofiban, Treprostinil, Cilostazol, Ticagrelor, Dipyridamole, Cilostazol, Streptokinase, Urokinase, Edoxaban, and Fondaparinux. Statin included Simvastatin, Lovastatin, Pravastatin, Fluvastatin, Atorvastatin, Rosuvastatin, and Pitavastatin.

### Statistical analysis

Propensity score matched pairs analyses were used to determine associations between renal diseases (CKD and ESRD) and the primary outcome (incident event of stroke). We developed a non-parsimonious multivariable logistic regression model to estimate propensity scores for CKD patients and ESRD patients. Clinical significance guided the initial choice of covariates in this model: age, sex, low income, hypertension, anemia, ischemic heart disease, diabetes, mental disorders, chronic obstructive pulmonary disease, heart failure, hyperlipidemia, traumatic brain injury, liver cirrhosis, atrial fibrillation, peripheral vascular disease, epilepsy, anticoagulant, anti-hypertension, and statin. We matched CKD patients and ESRD patients to control group without renal diseases using a greedy matching algorithm (without replacement) with a caliper width of 0.2 SD of the log odds of the estimated propensity score. Our study used chi-square tests to compare sociodemographic characteristics and coexisting medical conditions of cohorts with and without hemodialysis. We calculated the hazard ratios (HRs) with 95% confidence intervals (CIs) for risk of stroke associated with CKD and ESRD adjusting for age, sex, low income, coexisting diseases, and medications in multivariate Cox proportional hazards regression models. We calculated sex- and age-stratified analyses for the adjusted HRs of stroke associated with hemodialysis. In the nested cohort study, the baseline characteristics and coexisting medical conditions among stroke patients with and without hemodialysis were compared using chi-square tests. The adjusted rate ratios (RRs) and 95% CIs of post-stroke mortality associated with hemodialysis were calculated in multivariate Poisson regressions with adjustment for covariates in univariate analysis. The association between post-stroke mortality and hemodialysis was also analyzed in stratification by age and sex. SAS version 9.1 (SAS Institute Inc., Cary, NC, USA) statistical software was used for data analyses; two-sided *P* < 0.05 indicated significant differences between groups.

## Results

There were no significant differences in age and gender between people among CKD, ESRD and control patients under the frequency matching procedure used in this retrospective cohort study (Table 1). Proportionately more patients with CKD and ESRD than controls had low incomes, hypertension, anemia, ischemic heart disease, diabetes, mental disorders, heart failure, hyperlipidemia, liver cirrhosis, atrial fibrillation, peripheral vascular disease, epilepsy, or traumatic brain injury (all *p*-value <0.001). Cohorts with CKD and ESRD used more anticoagulants (p<0.0001), anti-hypertensive agents (p<0.0001), and statins (p<0.0001) compared with controls.

**Table 1:** Baseline characteristics for controls and patients with CKD and ESRD.

	Control (N = 11024)		CKD (N = 5512)		ESRD (N = 1378)		*P* value
Age, years	n	(%)	n	(%)	n	(%)	1.0000
20–29	368	(3.3)	184	(3.3)	46	(3.3)	
30–39	848	(7.7)	424	(7.7)	106	(7.7)	
40–49	2232	(20.3)	1116	(20.3)	279	(20.3)	
50–59	2280	(20.7)	1140	(20.7)	285	(20.7)	
60–69	2504	(22.7)	1252	(22.7)	313	(22.7)	
≥70	2792	(25.3)	1396	(25.3)	349	(25.3)	
Sex							1.0000
Female	5768	(52.3)	2884	(52.3)	721	(52.3)	
Male	5256	(47.7)	2628	(47.7)	657	(47.7)	
Low income	298	(2.7)	232	(4.2)	109	(7.9)	<0.0001
Medical conditions							
Hypertension	3895	(35.3)	2798	(50.8)	912	(66.2)	<0.0001
Anemia	1204	(10.9)	1309	(23.8)	880	(63.9)	<0.0001
Ischemic heart disease	1945	(17.6)	1864	(33.8)	600	(43.5)	<0.0001
Diabetes	1600	(14.5)	1522	(27.6)	571	(41.4)	<0.0001
Mental disorders	3258	(29.6)	2453	(44.5)	473	(34.3)	<0.0001
COPD	2482	(22.5)	2001	(36.3)	333	(24.2)	<0.0001
Heart failure	406	(3.7)	585	(10.6)	288	(20.9)	<0.0001
Traumatic brain injury	1035	(9.4)	729	(13.2)	169	(12.3)	<0.0001
Hyperlipidemia	1278	(11.6)	1072	(19.5)	162	(11.8)	<0.0001
Liver cirrhosis	517	(4.7)	514	(9.3)	139	(10.1)	<0.0001
Atrial fibrillation	306	(2.8)	291	(5.3)	94	(6.8)	<0.0001
Peripheral vascular disease	210	(1.9)	204	(3.7)	65	(4.7)	<0.0001
Epilepsy	92	(0.8)	84	(1.5)	34	(2.5)	<0.0001
Type of medication							
Anticoagulant	688	(6.2)	706	(12.8)	712	(51.7)	<0.0001
Anti-hypertension	2944	(26.7)	2204	(40.0)	667	(48.4)	<0.0001
Statin	2046	(18.6)	1668	(30.3)	465	(33.7)	<0.0001

COPD, chronic obstructive pulmonary disease; ESRD, end-stage renal disease.

After propensity-score matching, there were no significant differences in age, sex, low income, medical conditions, and medications between people among CKD (Table 2), ESRD (Table 3) and control group.

**Table 2:** Baseline characteristics for controls and CKD patients after matching with propensity score.

	Control (N = 2758)		CKD (N = 2758)		*P* value
Age, years	n	(%)	n	(%)	1.0000
20–29	138	(5.0)	138	(5.0)	
30–39	276	(10.0)	276	(10.0)	
40–49	682	(24.7)	682	(24.7)	
50–59	591	(21.4)	591	(21.4)	
60–69	543	(19.7)	543	(19.7)	
≥70	528	(19.1)	528	(19.1)	
Sex					1.0000
Female	1425	(51.7)	1425	(51.7)	
Male	1333	(48.3)	1333	(48.3)	
Low income	28	(1.0)	28	(1.0)	1.0000
Medical conditions					
Hypertension	1087	(39.4)	1087	(39.4)	1.0000
Mental disorders	1009	(36.6)	1009	(36.6)	1.0000
COPD	705	(25.6)	705	(25.6)	1.0000
Ischemic heart disease	480	(17.4)	480	(17.4)	1.0000
Diabetes	407	(14.8)	407	(14.8)	1.0000
Anemia	333	(12.1)	333	(12.1)	1.0000
Hyperlipidemia	323	(11.7)	323	(11.7)	1.0000
Traumatic brain injury	191	(6.9)	191	(6.9)	1.0000
Liver cirrhosis	87	(3.2)	87	(3.2)	1.0000
Heart failure	48	(1.7)	48	(1.7)	1.0000
Atrial fibrillation	27	(1.0)	27	(1.0)	1.0000
Peripheral vascular disease	8	(0.3)	8	(0.3)	1.0000
Epilepsy	3	(0.1)	3	(0.1)	1.0000
Type of medication					
Anti-hypertension	770	(27.9)	770	(27.9)	1.0000
Statin	514	(18.6)	514	(18.6)	1.0000
Anticoagulant	87	(3.2)	87	(3.2)	1.0000

COPD, chronic obstructive pulmonary disease

**Table 3:** Baseline characteristics for controls and ESRD patients after matching with propensity score.

	Control (N = 320)		ESRD (N = 320)		*P* value
Age, years	n	(%)	n	(%)	1.0000
20–29	9	(2.8)	9	(2.8)	
30–39	16	(5.0)	16	(5.0)	
40–49	60	(18.8)	60	(18.8)	
50–59	64	(20.0)	64	(20.0)	
60–69	72	(22.5)	72	(22.5)	
≥70	99	(30.9)	99	(30.9)	
Sex					1.0000
Female	161	(50.3)	161	(50.3)	
Male	159	(49.7)	159	(49.7)	
Low income	2	(0.6)	2	(0.6)	1.0000
Medical conditions					
Hypertension	163	(50.9)	163	(50.9)	1.0000
Anemia	120	(37.5)	120	(37.5)	1.0000
Ischemic heart disease	82	(25.6)	82	(25.6)	1.0000
Diabetes	75	(23.4)	75	(23.4)	1.0000
Mental disorders	78	(24.4)	78	(24.4)	1.0000
COPD	54	(16.9)	54	(16.9)	1.0000
Traumatic brain injury	18	(5.6)	18	(5.6)	1.0000
Hyperlipidemia	15	(4.7)	15	(4.7)	1.0000
Liver cirrhosis	13	(4.1)	13	(4.1)	1.0000
Heart failure	7	(2.2)	7	(2.2)	1.0000
Atrial fibrillation	3	(0.9)	3	(0.9)	1.0000
Peripheral vascular disease	0	(0.0)	0	(0.0)	1.0000
Epilepsy	0	(0.0)	0	(0.0)	1.0000
Type of medication					
Anti-hypertension	107	(33.4)	107	(33.4)	1.0000
Anticoagulant	54	(16.9)	54	(16.9)	1.0000
Statin	46	(14.4)	46	(14.4)	1.0000

COPD, chronic obstructive pulmonary disease; ESRD, end-stage renal disease.

**Fig 1:**
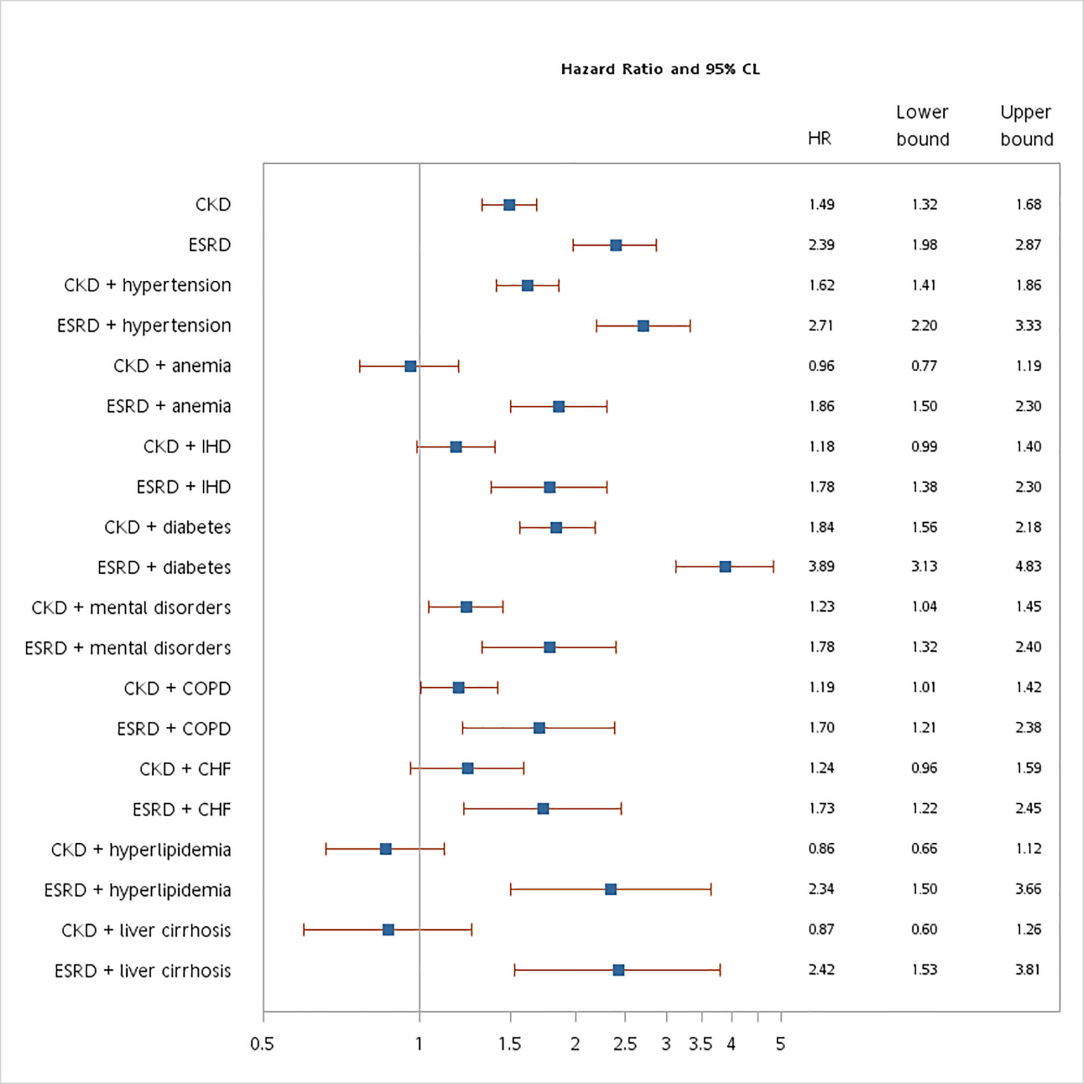
Incidence and risk of stroke for controls, CKD patients, and ESRD patients. CI, confidence interval; CHF, heart failure; ESRD, end-stage renal disease; HR, hazard ratio; IHD, ischemic heart disease. Incidence showed per 1000 person-years. Compared with control group (n = 11024), all hazard rations adjusted for all covariates listed in Table 1 in 17 multivariate Cox proportional hazard models. Under the consideration of death as a competing risk, the hazard ratios of stroke for CKD patients and ESRD patients were 1.45 (95% CI 1.27–1.66) and 2.22 (95% CI 1.79–2.76), respectively. After matching with propensity score by all covariates listed in Table 1, the hazard ratios of stroke for CKD patients and ESRD patients were 1.51 (95% CI 1.24–1.85) and 2.08 (95% CI 1.32–3.26), respectively. The interaction terms of ESRD*sex (p<0.0001), ESRD*anemia (p<0.0001), ESRD*ischemic heart disease (p<0.0001), ESRD*diabetes (p<0.0001), ESRD*mental disorders (p = 0.0002), ESRD*chronic obstructive pulmonary disease (p = 0.0021), ESRD*heart failure (p = 0.0020), ESRD*hyperlipidemia (p = 0.0002), ESRD*liver cirrhosis (p = 0.0002) were added in the multivariate Cox proportional hazard models.

Fig 1. shows the HR of stroke for patients with ESRD was 2.39 (95% CI 1.98–2.87) during the follow-up period compared with control cohort. The association between ESRD and risk of stroke was also significant in both sexes and all age groups analyzed: males (HR 2.73), females (HR 2.13), 20–39 years (HR 3.73), 40–49 years (HR 2.61), 50–59 years (HR 2.96), 60–69 years (HR 2.52), and ≥70 years (HR 1.68). Among ESRD patients, hypertension (HR 2.71), diabetes (HR 3.89), and liver cirrhosis (HR 2.42) were significant factors contributing to the risk of stroke. Patients with CKD had increased risk of stroke during the follow-up period (HR 1.49, 95% CI 1.32–1.68). Risk of stroke was associated with CKD in females (HR 1.52), males (HR 1.45), and every age group. Among patients with CKD, hypertension (HR 1.62) and diabetes (HR 1.84) increased risk of stroke.

After propensity-score matching, the HRs of stroke for patients with CKD and ESRD were 1.51 (95% CI 1.24–1.85) and 2.08 (95% CI 1.32–3.26), respectively, during the follow-up period (Table 4).

**Table 4:** Incidence and risk of stroke for CKD and ESRD patients and controls.

	n	Person-years	Stroke events	Incidence*	HR	(95% CI)†
Controls without CKD	2758	29827	168	5.63	1.00	(reference)
CKD patients	2758	28816	237	8.22	1.51	(1.24–1.85)
Controls without ESRD	320	3347	30	8.96	1.00	(reference)
ESRD patients	320	2850	52	18.2	2.08	(1.32–3.26)

CI, confidence interval; CKD, chronic kidney disease; ESRD, end-stage renal disease; HR, hazard ratio

*Per 1000 person-years.

†Adjusted all covariates listed in Table 1 in the multivariate Cox’s proportional hazard models.

Table 5 shows baseline characteristics of newly diagnosed stroke patients among CKD, ESRD and control cohorts. Compared with controls, ESRD was associated with higher proportions of females (*p*<0.0001), low income (*p*<0.0001), hemorrhagic stroke (*p*<0.0001), diabetes (*p*<0.0001), ischemic heart disease (*p*<0.0001), chronic obstructive pulmonary disease (*p*<0.0001), heart failure (*p*<0.0001), anemia (*p*<0.0001), hyperlipidemia (*p*<0.0001), traumatic brain injury (*p* = 0.0497), liver cirrhosis (*p*<0.0001), peripheral vascular disease (*p*<0.0001), and epilepsy (*p*<0.0001). This ESRD group also used more medications than controls, specifically anti-hypertension drugs (*p*<0.0001), statins (*p*<0.0001), and anticoagulants (*p*<0.0001). Stroke patients with CKD had higher proportions of older people, other stroke subtypes, hypertension, diabetes, ischemic heart disease, mental disorders, chronic obstructive pulmonary disease, heart failure, anemia, hyperlipidemia, traumatic brain injury, atrial fibrillation, and peripheral vascular disease compared with stroke controls (all *p*<0.0001).

**Table 5:** Baseline characteristics of hospitalized stroke patients with CKD and ESRD.

	Stroke controls (N = 300,300)		Stroke with CKD (N = 12340)		Stroke with ESRD (N = 5998)		*P* value
Gender	n	(%)	n	(%)	n	(%)	<0.0001
Female	124,440	(41.4)	4973	(40.3)	3093	(51.6)	
Male	175,860	(58.6)	7367	(59.7)	2905	(48.4)	
Age							<0.0001
20–29	2473	(0.8)	34	(0.3)	26	(0.4)	
30–39	8198	(2.7)	156	(1.3)	101	(1.7)	
40–49	27,849	(9.3)	559	(4.5)	498	(8.3)	
50–59	53,369	(17.8)	1458	(11.8)	1360	(22.7)	
60–69	69,938	(23.3)	2542	(20.6)	1735	(28.9)	
70–79	87,125	(29.0)	4667	(37.8)	1672	(27.9)	
≥80	51,348	(17.1)	2924	(23.7)	606	(10.1)	
Low income	15,160	(5.1)	721	(5.8)	460	(7.7)	<0.0001
Urbanization							0.0015
Low	3748	(1.3)	138	(1.1)	67	(1.1)	
Moderate	85,473	(28.5)	3461	(28.1)	1577	(26.3)	
High	211,079	(70.3)	8741	(70.8)	4354	(72.6)	
Teaching hospital	271,636	(90.5)	10911	(88.4)	5542	(92.4)	<0.0001
Ownership of hospital							<0.0001
Private	224604	(74.8)	8847	(71.7)	4514	(75.3)	
Public	75696	(25.2)	3493	(28.3)	1484	(24.7)	
Level of hospital							<0.0001
Medical center	91588	(30.5)	3935	(31.9)	2023	(33.7)	
Regional hospital	142557	(47.5)	5394	(43.7)	2832	(47.2)	
Local hospital	66155	(22.0)	3011	(24.4)	1143	(19.1)	
Type of stroke							<0.0001
Hemorrhagic	68,799	(22.9)	2346	(19.0)	2062	(34.4)	
Ischemic	179,443	(59.8)	7379	(59.8)	3106	(51.8)	
Others	52,058	(17.3)	2615	(21.2)	830	(13.8)	
Coexisting diseases							
Hypertension	142,388	(47.4)	7715	(62.5)	2809	(46.8)	<0.0001
Diabetes	67,751	(22.6)	4308	(34.9)	2732	(45.6)	<0.0001
Ischemia heart disease	40,606	(13.5)	3197	(25.9)	1586	(26.4)	<0.0001
Mental disorder	49,641	(16.5)	3086	(25.0)	1021	(17.0)	<0.0001
COPD	46,501	(16.5)	3348	(27.1)	833	(13.9)	<0.0001
Heart failure	10,356	(3.5)	1214	(9.8)	627	(10.5)	<0.0001
Anemia	6383	(2.1)	1093	(8.9)	582	(9.7)	<0.0001
Hyperlipidemia	19,433	(6.5)	1399	(11.3)	565	(9.4)	<0.0001
Traumatic brain injury	14,261	(4.8)	762	(6.2)	326	(5.4)	<0.0001
Liver cirrhosis	3700	(1.2)	271	(2.2)	153	(2.6)	<0.0001
Atrial fibrillation	5061	(1.7)	365	(3.0)	116	(1.9)	<0.0001
PVD	3091	(1.0)	263	(2.1)	116	(1.9)	<0.0001
Epilepsy	1076	(0.4)	34	(0.3)	40	(0.7)	0.0001
Types of medication							
Anti-hypertension	62802	(20.9)	4123	(33.4)	2053	(34.2)	<0.0001
Statin	37637	(12.5)	3106	(25.2)	1633	(27.2)	<0.0001
Anticoagulant	7343	(2.5)	545	(4.4)	448	(7.5)	<0.0001

COPD, chronic obstructive pulmonary disease; CKD, chronic kidney disease; ESRD, end-stage renal disease; PVD, peripheral vascular disease.

Thirty-day post-stroke mortality associated with CKD was (RR 1.44, 95% CI 1.33–1.56). This association was significant in females (HR 1.49), males (HR 1.41), and every age group (Table 6). Among hospitalized stroke patients, the RR of post-stroke mortality in ESRD cohort was 2.62 (95% CI 2.43–2.82) compared with control group. Post-stroke mortality was associated with hemodialysis in male (RR 2.56) and female (RR 2.67) stroke patients. The corresponding association of mortality was also significant in stroke patients aged 20–49 years (RR 2.64), 50–59 years (RR 3.03), 60–69 years (RR 2.63), 70–79 years (RR 2.44), and ≥80 years (RR 2.01). Among stroke patients with CKD or ESRD, anemia (HR 1.94 vs. 3.34), ischemic heart disease (HR 1.50 vs. 3.12), diabetes (HR 1.49 vs. 3.24), heart failure (HR 1.79 vs. 2.33), and liver cirrhosis (HR 2.24 vs. 2.64) were contributors to 30-day post-stroke mortality.

**Table 6:** Thirty-day post-stroke mortality for patients with CKD and ESRD.

	n	deaths	mortality, %	RR	(95% CI)*
Control	300,300	10,129	3.4	1.00	(reference)
CKD†	12340	594	4.8	1.42	(1.31–1.54)
ESRD†	5998	667	11.1	2.58	(2.39–2.78)
CKD + hypertension†	7715	357	4.6	1.39	(1.26–1.54)
ESRD + hypertension†	2809	283	10.1	2.23	(1.99–2.50)
CKD + anemia†	1093	78	7.1	1.83	(1.49–2.26)
ESRD + anemia†	582	69	11.9	2.67	(2.14–3.34)
CKD + IHD†	3197	145	4.5	1.46	(1.24–1.71)
ESRD + IHD†	1586	166	10.5	2.49	(2.15–2.89)
CKD + diabetes†	4308	185	4.3	1.37	(1.19–1.58)
ESRD + diabetes†	2732	291	10.7	2.49	(2.23–2.78)
CKD + mental disorders†	3086	132	4.3	1.24	(1.04–1.47)
ESRD + mental disorders†	1021	122	12.0	2.57	(2.18–3.03)
CKD + COPD†	3348	141	4.2	1.24	(1.05–1.46)
ESRD + COPD†	833	89	10.7	2.46	(2.03–2.99)
CKD + heart failure†	1214	97	8.0	1.60	(1.21–2.13)
ESRD + heart failure†	627	45	7.2	2.15	(1.77–2.61)
CKD + hyperlipidemia†	1399	38	2.7	0.99	(0.73–1.35)
ESRD + hyperlipidemia†	565	54	9.6	2.36	(1.83–3.04)
CKD + liver cirrhosis†	271	25	9.2	1.91	(1.32–2.77)
ESRD + liver cirrhosis†	153	19	12.4	2.09	(1.39–3.16)
Female					
Control	124,440	3871	3.1	1.00	(reference)
CKD	227	227	4.6	1.47	(1.29–1.67)
ESRD	3093	319	10.3	2.62	(2.35–2.93)
Male					
Control	175,860	6258	3.6	1.00	(reference)
CKD	7367	367	5.0	1.39	(1.25–1.54)
ESRD	2905	348	12.0	2.54	(2.30–2.81)
20–49 years					
Control	38,520	2095	5.4	1.00	(reference)
CKD	749	66	8.8	1.84	(1.46–2.31)
ESRD	625	112	17.9	2.60	(2.18–3.10)
50–59 years					
Control	53,369	1691	3.2	1.00	(reference)
CKD	1458	70	4.8	1.59	(1.27–2.00)
ESRD	1360	192	14.1	3.01	(2.61–3.49)
60–69 years					
Control	69,938	1500	2.1	1.00	(reference)
CKD	2542	80	3.2	1.38	(1.11–1.73)
ESRD	1735	144	8.3	2.60	(2.19–3.09)
70–79 years					
Control	87,125	2352	2.7	1.00	(reference)
CKD	4667	181	3.9	1.35	(1.17–1.57)
ESRD	1672	149	8.9	2.46	(2.10–2.89)
≥80 years					
Control	51,348	2491	4.9	1.00	(reference)
CKD	2924	197	6.7	1.34	(1.17–1.55)
ESRD	606	70	11.6	2.03	(1.63–2.54)

CI, confidence interval; CKD, chronic kidney disease; IHD, ischemic heart disease; ESRD, end-stage renal disease; RR, rate ratio.

*Adjusted for all covariates listed in Table 3 in 17 multivariate Cox proportional hazard models.

†Compared with the control group (n = 300,300).

## Discussion

Our nationwide retrospective cohort study showed patients with CKD or ESRD had nearly 1.5 to 2.5-fold risk of stroke respectively when compared with controls. Higher risk of stroke was noted when CKD or ESRD patients also had hypertension or diabetes. The nested cohort study showed CKD or ESRD patients with anemia, diabetes, ischemic heart disease or liver cirrhosis had higher post-stroke mortality than controls. In CKD patients, post-stroke mortality was highest in patients with liver cirrhosis. Mental disorders, COPD, and hyperlipidemia affected post-stroke death rates only in ESRD patients.

Hypertension, diabetes, and other traditional cardiovascular risk factors such as aging, hyperlipidemia, obesity, and smoking [2–4,19–21] are the main causes of renal function impairment in many countries [1]. In our investigation, CKD or ESRD patients with hypertension or diabetes exhibited higher risk of stroke. Uremia represents a risk complex with multiple etiologies and comorbidities associated with vascular change and dialysis-related problems [17,22–23]. Although traditional stroke risk factors are more prevalent in uremic patients than in the general population, the increased risk of stroke observed in uremic patients could not be explained solely by higher comorbidity prevalence. As CKD has been recognized as a significant predictor for stroke beyond conventional cardiovascular risk factors [3,14,15,24,25], the long-term effects of end-stage renal disease itself and renal replacement therapy were categorized as non-traditional risk factors, including vessel calcification, uremic toxins, frequent vascular access, and anticoagulant use during dialysis [4,26]. These uremic-specific risk factors might induce endothelial dysfunction, platelet aggregation, and vascular calcification that increase cerebrovascular disease risks, an effect amplified if CKD progresses to ESRD [2,7]. Similarly, cerebral and renal function are more vulnerable to vascular injury and arteriosclerotic insult with endothelial function impairment, ischemic arteriosclerosis, inadequate perfusion, neurovascular coupling, and blood-brain barrier disruption [2].

The exact mechanism of stroke in CKD and uremic patients remains unclear. Intracranial artery calcification, blood pressure control, and associated medical comorbidities are possible explanations [27,28]. Furthermore, the Klotho protein was considered as a regulator of cardiovascular disease, and the progress of kidney dysfunction decreases Klotho gene expression, which might in turn impair calcium and phosphate metabolism and cell function [2]. Nutritional status is another stroke risk concern in chronic dialysis patients, whose serum albumin levels have been found to be lower than normal [3,26,29]. Hypoalbuminemia results in red blood cell deformity and endothelial cell dysfunction [29]. Cerebral blood flow and cerebral oxygenation also decreases significantly in ESRD patients, predisposing them to brain atrophy, cognitive dysfunction, and stroke [30]. Other possible reasons for ischemic stroke include erythropoietin-induced thromboembolic events [12,26,31], arteriovenous shunt-related steal-like influence [12], and hypotensive effect during the ultra-filtration stage of hemodialysis [1]. For hemorrhagic stroke, hypertension and routine use of heparin as anticoagulant during hemodialysis are considered major determinants of this complication [1,8,15]. Other possible risk factors for hemorrhagic stroke include impaired platelet function and polycystic kidney [3,30,31].

Comorbidities’ differential impact on stroke incidence and post-stroke mortality between CKD and ESRD was an important issue that was not well investigated in previous studies. Among all listed risk factors, anemia, hyperlipidemia, and liver cirrhosis did not exhibit significant influence on stroke incidence in patients with CKD, but showed some effect in patients with ESRD. Different degrees of kidney dysfunction severity and various effects of treatment for coexisting clinical conditions may account for these discrepancies.

As hyperlipidemia is one of the traditional atherosclerotic risk factors in CKD, control of dyslipidemia might not only decrease cardiovascular events, but also delay the progression of kidney disease [32]. In our study, hyperlipidemia did not influence stroke incidence and post-stroke mortality significantly in patients with CKD, but had some effect in patients with ESRD. This might be attributed to higher frequency of statin use in CKD and ESRD populations. Stain had shown benefits in improving triglyceride levels, elevated low-density lipoprotein and low high-density lipoprotein in patients with CKD not requiring dialysis therapy [32]. Anemia is a common complication of CKD and might reach peaks when renal dysfunction progresses to ESRD [33–34]. Reduced oxygen transport and supply might cause cerebral ischemia and compensatory increase in cerebral blood flow. The effect of dilutional anemia in increasing cerebral blood flow was predominately higher in patients with ESRD [35]. The postulated mechanism can be attributed to vasodilation in response to reduced arterial oxygen tension or decreased blood viscosity [35,36]. Although elevated CBF may compensate for anemia-induced hypoxia, cerebral oxygen delivery was still relatively low [35]. Furthermore, cerebrovascular response to CO2 was obviously attenuated in anemic individuals with chronic renal failure, which resulted in decreased cerebral vasodilatory reserve [36]. Varying degrees of anemia and compensatory response between CKD and ESRD might explain different impacts on stroke incidence. Reduced prevalence of cardiovascular complications in patients with cirrhosis had been reported in previous studies [37]. Impaired hemostatic and platelet function might act as a protective mechanism against ischemic events in cirrhotic patients, possibly due to decreased prevalence of abnormal supraortic flow patterns in cirrhotic populations [37]. In the present study, liver cirrhosis did not show significant influence on stroke incidence in patients with CKD, and exhibited similar hazard ratio to that of ESRD. Decreased coagulation components and reduced hemostatic function could account for this. Nevertheless, post-stroke mortality in CKD patients was highest when liver cirrhosis existed concurrently. Further investigation is needed to determine whether type and etiology of cirrhosis and its related pathophysiological consequences contribute to post-stroke deaths.

Chronic obstructive pulmonary disease (COPD) and mental disorders did not increase incidence of stroke in CKD and ESRD patients, but showed obvious additive effects on post-stroke mortality. COPD is characterized by chronic inflammatory response, and is a strong risk factor for CKD development and progression [38]. They share common atherosclerotic risk factors through systemic inflammatory processes such as hypertension and diabetes [38]. The possible mechanism for developing COPD was attributed to the existence of transforming growth factor (TGF)-β and inflammatory cytokine tumor necrosis factor-alpha [38]. Both mediators are seen as possible pathogens capable of causing atherosclerotic change in patients with COPD or CKD [38]. Though the assumption will still need to be confirmed, the discrepancy between stroke incidence and post-stroke mortality in patients with ESRD might be due to differences in disease severity and inflammatory response. Patients with CKD often showed global cognitive function decline and dementia as renal function deteriorated [39,40]. Cognitive dysfunction can have multiple causes that effect metabolic and biochemical derangements in the central nervous system, including secondary hyperparathyroidism, amino acid abnormality, and uremic-induced neurotoxicity [40]. When kidney function deteriorates to ESRD, these pathological effects can increase post-stroke mortality when combined with mental disorders.

When anemia, diabetes, and IHD existed concurrently, post-stroke mortality was especially high in patients with either CKD or ESRD. To treat concurrent anemia, erythropoiesis-stimulating agents (ESAs) have been a main strategy to correct anemia since 1987 [33,34]. Although such therapy may benefit CKD patients by improving quality of life and reducing transfusion frequency, it has been associated with increased risk of stroke, thrombosis, and mortality, especially among individuals with cancer and populations treated with higher doses of ESAs [33,34]. Though our study lacked information on ESA use, this therapy is still postulated as the most likely reason for higher mortality in anemic patients with CKD or ESRD. Substantial effort must be devoted to achieving appropriate hemoglobin levels with ESA therapy. Diabetes constitutes the major cause of renal failure [1], and the impact of diabetes on stroke incidence and post-stroke survival in patients with CKD or ESRD should be emphasized in further research. Diabetes and ESRD have synergistic influences on cardiovascular risks such as acute myocardial complication, heart failure, and stroke [7]. The pathophysiological explanations for these phenomena include the combined effects of elevated blood pressure, dyslipidemia, endothelial dysfunction, oxidative stress, insulin resistance, and vascular local inflammation signaling that leads to atherosclerosis and increases the likelihood of stroke [7]. Cardiovascular disease and CKD constitute a vicious cycle promoting each other’s occurrence and severity. This mechanism is complex, with overlapping interrelationships including upregulation of the renin-angiotensin-aldosterone axis and sympathetic nervous system in the cardiovascular system as well as fluid overloading, heart remodeling, impaired blood pressure control, and inflammatory response in patients with renal dysfunction [41]. In this study, ischemic heart disease and heart failure did not show additive effects on stroke incidence in patients with CKD and ESRD, perhaps because of pharmacological interventions. Anti-platelet medications, anti-coagulation drugs, NSAIDs, angiotensin converting enzyme inhibitors, calcium channel blockers, and statins were effective in reducing cardiovascular risks in patients with CKD and ESRD [33,42–44]. Yet once stroke occurred, ischemic heart disease elevated post-stroke mortality in patients with ESRD, possibly because of the synergistic influences of cardiovascular disease and renal function impairment.

The strength of this investigation was cohort study design, larger sample size, multivariate adjustment of confounding factors, and reporting various types of stroke. However, we admit some limitations that should be considered when interpreting the results of this study. First, our study lacks information on detailed sociodemographics; on lifestyle factors like smoking, alcohol intake, nutrition and physical activity levels; on severity and duration of kidney dysfunction; on hemodialysis compliance characteristics; on changes in hemodynamic variables change; on anticoagulant management; and on results of clinical examinations. There also is a risk that we underestimated the prevalence of coexisting medical conditions and incidence of stroke because patients with minor illness might neglect to seek medical care. Third, the severity of stroke was not definedm and this might bias risk estimations associating CKD and ESRD with post-stroke mortality. Finally, residual confounding is always possible despite controlling for various possible confounders in the regression models.

In conclusion, our analysis provided comprehensive evaluation of stroke risk and post-stroke mortality in patients with CKD and ESRD and established that these renal disorders were independent risk factors for both outcomes. Further investigation is necessary to develop strategies to reduce stroke risk and post-stroke mortality in these high-risk patient populations.
